# Microenvironment heterogeneity affected by anthropogenic wildfire-perturbed soil mediates bacterial community in *Pinus tabulaeformis* forests

**DOI:** 10.3389/fmicb.2024.1415726

**Published:** 2024-07-09

**Authors:** Guanhong Liu, Ze Gu, Xiaodong Liu, Bingyi Li

**Affiliations:** ^1^School of Ecology and Nature Conservation, Beijing Forestry University, Beijing, China; ^2^Hebei Normal University, Shijiazhuang, Hebei, China

**Keywords:** anthropogenic wildfire, microenvironment heterogeneity, biodiversity, soil nutrients, bacterial community

## Abstract

**Introduction:**

In recent years, the frequency and intensity of anthropogenic wildfires have drastically increased, significantly altering terrestrial ecosystems worldwide. These fires not only devastate vegetative cover but also impact soil environments and microbial communities, affecting ecosystem structure and function. The extent to which fire severity, soil depth, and their interaction influence these effects remains unclear, particularly in *Pinus tabulaeformis* forests.

**Methods:**

This study investigated the impact of wildfire intensity and soil stratification on soil physicochemical properties and microbial diversity within *P. tabulaeformis* forests in North China. Soil samples were collected from different fire severity zones (Control, Light, Moderate, High) and depths (topsoil: 0–10 cm; subsoil: 10–20 cm). Analyses included measurements of soil pH, organic carbon (SOC), total nitrogen (TN), and other nutrients. Microbial diversity was assessed using 16S rRNA gene sequencing.

**Results:**

Our findings revealed significant variations in soil pH, SOC, TN, and other nutrients with fire severity and soil depth, profoundly affecting microbial community composition and diversity. Soil pH emerged as a critical determinant, closely linked to microbial α-diversity and community structure. We found that fire severity significantly altered soil pH (*p* = 0.001), pointing to noteworthy changes in acidity linked to varying severity levels. Topsoil microbial communities primarily differentiated between burned and unburned conditions, whereas subsoil layers showed more pronounced effects of fire severity on microbial structures. Analysis of bacterial phyla across different fire severity levels and soil depths revealed significant shifts in microbial communities. Proteobacteria consistently dominated across all conditions, indicating strong resilience, while *Acidobacteriota* and *Actinobacteriota* showed increased abundances in high-severity and light/moderate-severity areas, respectively. *Verrucomicrobiota* were more prevalent in control samples and decreased significantly in fire-impacted soils. *Chloroflexi* and *Bacteroidota* displayed increased abundance in moderate and high-severity areas, respectively. Correlation analyses illustrated significant relationships between soil environmental factors and dominant bacterial phyla. Soil organic carbon (SOC) showed positive correlations with total nitrogen (TN) and alkaline hydrolysable nitrogen (AN). Soil pH exhibited a negative correlation with multiple soil environmental factors. Soil pH and available phosphorus (AP) significantly influenced the abundance of the phylum *Myxococcota*. Soil water content (WC) significantly affected the abundances of *Acidobacteriota* and *Actinobacteriota*. Additionally, ammonium nitrogen (NH_4_^+^-N) and nitrate nitrogen (NO_3_^−^-N) jointly and significantly impacted the abundance of the phylum *Chloroflexi*.

**Discussion:**

This study highlights the significant long-term effects of anthropogenic wildfires on soil microenvironment heterogeneity and bacterial community structure in *P. tabulaeformis* forests in North China, 6 years post-fire. Our findings demonstrate that fire severity significantly influences soil pH, which in turn affects soil nutrient dynamics and enhances microbial diversity. We observed notable shifts in the abundance of dominant bacterial phyla, emphasizing the critical role of soil pH and nutrient availability in shaping microbial communities. The results underscore the importance of soil stratification, as different soil layers showed varying responses to fire severity, highlighting the need for tailored management strategies. Future research should focus on long-term monitoring to further elucidate the temporal dynamics of soil microbial recovery and nutrient cycling following wildfires. Studies investigating the roles of specific microbial taxa in ecosystem resilience and their functional contributions under varying fire regimes will provide deeper insights. Additionally, exploring soil amendments and management practices aimed at optimizing pH and nutrient availability could enhance post-fire recovery processes, supporting sustainable ecosystem recovery and resilience.

## Introduction

Wildfires are a natural and transformative force in forest ecosystems worldwide, shaping biodiversity, altering soil properties, and influencing nutrient cycling (Poulos et al., 2021; Zhao et al., 2023). The frequency, severity, and extent of wildfires have been profoundly affected by climate change and human activities, leading to significant ecological and environmental impacts (Poulos et al., 2021; Lopez et al., 2023; Qin et al., 2023). However, the role of wildfires extends beyond immediate destruction (Certini, 2005). They play a critical role in ecological succession, nutrient redistribution, and the regeneration of forest ecosystems (Tang et al., 2022; Qin et al., 2023). Wildfires can modify soil properties, including pH levels, soil organic matter, and microbial community composition, thereby influencing the post-fire recovery trajectory of these ecosystems (Lisa et al., 2016; Salgado et al., 2024). As the population rapidly grows, along with the increase in human activities and the demand for economic development, the ecological environment has been continually degraded. Human activities such as burning paper during ancestral grave visits and smoking in parks have led to an increase in the number of human-caused forest fires (Marey-Perez et al., 2023; Vachula et al., 2023). These fires disrupt the internal balance of forest ecosystems, increasing entropy and pushing ecosystems into a state of chaos and disorder (Su et al., 2021; Song Z et al., 2021; Qin et al., 2023).

Recent studies illustrate the complex dynamics of soil microbial communities in response to wildfires and subsequent management interventions. Research by Yang et al. alongside García-Carmona et al., highlights significant shifts in microbial community composition post-wildfire, with minimal management impacts and potential biocrust-mediated recovery, respectively (Garcia-Carmona et al., 2022; Yang et al., 2024). Divergent microbial recovery trajectories in boreal forests as demonstrated by Porter et al. and the critical interplay of soil carbon and nitrogen highlighted by Hopkins et al. underscore the multifaceted nature of microbial responses to environmental disturbances (Porter et al., 2023; Hopkins et al., 2024). Further, the combined effects of drought legacies and wildfires on microbial dynamics, as explored by Dai et al. emphasize the importance of considering historical climatic conditions (Dai et al., 2021). Together, these studies reinforce the need for nuanced fire management and ecological restoration strategies that account for the diverse responses of microbial communities to fire disturbances.

*Pinus tabulaeformis*, a characteristic conifer species in North China, is notable for its substantial resin content, classifying it as a highly flammable species (Xu L et al., 2022; Qi and Yin, 2023). Previous research has extensively explored the immediate and long-term effects of wildfires on various forest types. Studies have shown that wildfires can lead to significant changes in soil chemical properties, alter microbial diversity, and impact nutrient availability. However, the interplay between fire severity, soil depth, and their combined effects on soil properties and microbial communities, particularly in *P. tabulaeformis* forests, remains less understood. This research specifically targets the complex interactions between fire severity, soil depth, and their cumulative effects on soil properties and microbial communities within *P. tabulaeformis* forests. Given the high flammability and ecological significance of *P. tabulaeformis*, understanding these dynamics is crucial for effective forest management and recovery strategies post-wildfire. The aim of this study is to meticulously examine how varying fire intensities influence soil physicochemical properties across different soil depths and to determine how these alterations affect microbial community composition and diversity. By providing a detailed analysis of soil microenvironment heterogeneity and microbial community structure response to fire, this study seeks to delineate the underlying mechanisms of microbial succession post-disturbance. The findings are expected to offer valuable insights into the resilience and long-term ecological functions of forest ecosystems, thereby informing targeted approaches for the rehabilitation and management of fire-affected areas.

## Materials and methods

### Study area

In April 2015, the forest fire occurred in Dawopu Village, Liuxi Town, Pingquan County, Chengde City, Hebei Province, China. The ignition was caused by local villagers burning paper during ancestral grave visits, leading to the wildfire spreading over an area approximately 53.33 hectares. The fire affected a natural secondary forest predominantly composed of *P. tabulaeformis*, with the forest stand experiencing varying degrees of fire disturbance. Subsequently, the area was left without human interference, providing a unique opportunity to study the impact of arson-related factors on soil nutrients and microbial communities in the affected region. This context underscores the significance of assessing how human-induced fire events influence ecological processes and soil dynamics.

### Plots setting and samples collection

**Figure 1:**
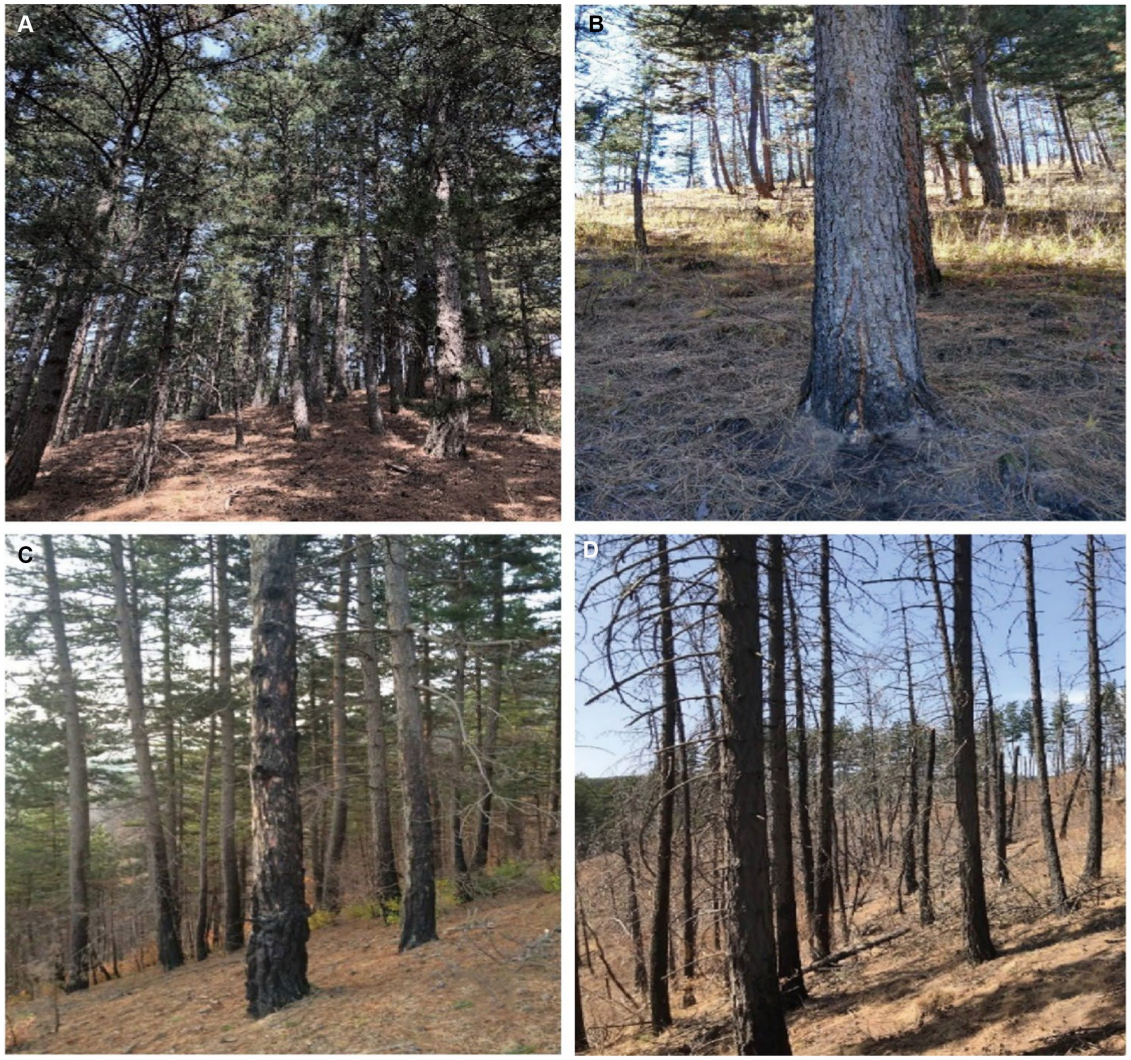
Photographs depicting different fire severity areas. **(A)** control test area, **(B)** light severity area, **(C)** moderate severity area, **(D)** high severity area.

Plots were set after fire in 2015. Criteria for categorizing fire intensities were based on the extent of tree blackening and tree mortality rates (Niccoli et al., 2019). Light-severity fires were identified by blackened heights of ≤3 m and tree mortality rates of ≤10%. In contrast, high-severity fires were characterized by blackened heights of ≥6 m, tree mortality rates of ≥70%, severe destruction of ground vegetation, and noticeable changes in soil color and structure (Niccoli et al., 2019). Additionally, there is a moderate-severity zone located between the light-severity and high-severity areas. All the plots are as follows (Figure 1).

Fires exhibiting characteristics between these extremes were classified as moderate-intensity. Control plots were selected from unburnt areas that shared similar geographical conditions. For each fire severity level, three replicate plots were established, each measuring 20 m × 20 m, totaling 12 plots. Detailed information is provided in Table 1.

**Table 1:** Sample plots data collection.

Fire severity	Plot number	Tree height (m)	DBH (cm)	Canopy coverage	Blackened height (m)	Tree mortality (%)
	C1	7.20	12.00	0.85	0.00	0.00
Control test (C)	C2	5.30	8.70	0.80	0.00	0.00
	C3	6.40	8.80	0.82	0.00	0.00
	L1	5.30	12.00	0.75	0.42	13.26
Light (L)	L2	5.00	8.20	0.72	0.50	18.20
	L3	7.40	13.20	0.70	0.67	16.35
	M1	15.80	17.20	0.69	4.70	54.60
Moderate (M)	M2	7.10	8.60	0.71	3.70	63.46
	M3	8.60	12.60	0.70	3.95	66.72
	H1	8.50	16.30	<0.10	8.50	100.00
High (H)	H2	9.20	11.90	<0.10	9.20	100.00
	H3	11.40	12.80	<0.10	11.40	100.00

In this study, soil samples were collected using a soil auger with a diameter of 37 mm from April 19 to April 24, 2021. To account for the variability introduced by different fire intensities, a five-point sampling method was employed within each plot, ensuring that three replicate samples were taken at each point. The process involved initially removing surface litter, then utilizing the soil drill to extract samples at designated depths of 0–10 cm (top soil layer, T) and 10–20 cm (sub soil layer, S), recognizing that the impact of the fire, which occurred over 6 years prior, might be predominantly observed in these surface or shallow layers except in areas of high fire severity. After collection, samples from the five points within a plot were homogeneously mixed to form a composite sample, with the fresh weight recorded prior to sealing in ziplock bags for preservation. In total, 24 soil samples were collected, comprising 12 from the top layer and 12 from the sub-layer, prepared for both physical and chemical properties analysis and air-drying. A separate portion of these samples was allocated for 16S rRNA analysis, necessitating storage at a constant low temperature of −20°C to preserve the integrity for microbiological assessments. This meticulous sampling strategy, considering depth and fire intensity variations, provides a robust framework for analyzing the post-fire soil dynamics and microbial community structures within the affected *P. tabulaeformis* forests.

### DNA extraction

Soil samples were collected using a soil auger with a diameter of 37 mm from each plot. The samples were stored at −20°C until further processing. DNA was extracted from 0.5 g of soil using the FastDNA™ SPIN Kit for Soil (MP Biomedicals, Solon, OH, United States) according to the manufacturer’s protocol. This kit employs a combination of mechanical and chemical lysis to efficiently lyse bacterial cells and release their DNA. The extracted DNA was then quantified using a NanoDrop 2000 spectrophotometer (Thermo Fisher Scientific, Waltham, MA, USA) and checked for purity and integrity by 1% agarose gel electrophoresis.

### PCR amplification

The V3-V4 hypervariable regions of the bacterial 16S rRNA genes were amplified by PCR using the extracted DNA (Zeng and An, 2021). The amplification protocol included an initial denaturation at 95°C for 5 min, followed by 33 cycles of denaturation at 95°C for 30 s, annealing at 56°C for 30 s, and extension at 72°C for 40 s, with a final extension at 72°C for 10 min. Universal primers 338F (5’-ACTCCTACGGGAGGCAGCAG-3′) and 806R (5’-GGACTACHVGGGTWTCTAAT-3′), each containing an 8-nucleotide barcode unique to each sample, were used. The PCR reactions were performed in a 50 μL mixture containing 5 μL of 10× Pyrobest Buffer, 4 μL of 2.5 mM dNTPs, 2 μL of each primer (10 μM), 0.3 μL of Pyrobest DNA Polymerase (2.5 U/μL; TaKaRa DR005A), and 30 ng of template DNA.

The PCR products were detected by 1% agarose gel electrophoresis and purified using the Agencourt AMPure XP nucleic acid purification kit (Beckman Coulter, Brea, CA, United States). The classification of Operational Taxonomic Units (OTUs) was based on a 97% similarity criterion. The Uclust consensus taxonomy assigner was employed for alignment analysis of OTU representative sequences and to annotate species information across various community levels. MiSeq PE300 library construction was commissioned by Beijing Allwegene Technology Co. Ltd., China.

### Processing of sequencing data

Initial sequencing data processing was conducted using the QIIME software package (Quantitative Insights into Microbial Ecology, version 1.8.0).^1^ Raw sequences were selected based on criteria such as sequence length, quality, and the presence of primers and tags. The sequences were quality-filtered by trimming reads at any site receiving an average quality score below 20 over a 10 bp window, discarding reads shorter than 50 bp after trimming. Sequences required exact barcode matching, allowed up to two nucleotide mismatches in primer sequences, and reads containing ambiguous characters were removed. Sequences were assembled based on overlaps exceeding 10 bp, with unassembled reads discarded. Chimeric sequences were identified and removed using the UCHIME algorithm (Edgar et al., 2011). Subsequently, sequences were clustered into operational taxonomic units (OTUs) at a 97% similarity threshold using UCLUST (Edgar, 2010), with each OTU taxonomically annotated against the Silva 138 16S rRNA database at a 90% confidence threshold (Quast et al., 2013). OTUs represented by fewer than five reads were excluded to minimize artifacts potentially inflating species richness due to sequencing errors.

### Bacterial community analysis

Bacterial community analysis was primarily divided into two parts: α-diversity and β-diversity. α-Diversity refers to the variety and abundance of species within a particular area or ecosystem. It provides insights into the complexity of microbial communities within a single sample. The metrics selected for α-diversity analysis in this study include the Chao1 index, PD_whole_tree, and the Shannon index (Chernov et al., 2015). Additionally, the redundancy analysis (RDA) is performed based on these metrics. Chao1 provides an insight into the total diversity contained within a sample. Shannon index estimates the microbial diversity within samples. A higher Shannon value indicates greater community diversity, encompassing both abundance and evenness of species. PD_whole_tree is phylogenetic diversity within the whole tree takes into account both the abundance of species and their evolutionary distances (Zhu et al., 2024). This diversity index is calculated based on a phylogenetic tree constructed from the representative sequences of OTUs in each sample. The summation of the branch lengths of all representative sequences in a sample gives a value that correlates with community diversity. The greater the value, the higher the community diversity.

For β-diversity analysis, Nonmetric Multidimensional Scaling (NMDS) is selected due to its effectiveness in simplifying complex data while maintaining sample relationships (Abutaha et al., 2021). NMDS aids in visualizing ecological variances and connections among microbial communities, enhancing insights into compositional shifts across varied environments or treatments.

### Soil environmental factors

The correlation between bacterial communities and the soil environmental factors was meticulously examined in this study. The environmental factors of soil analyzed include Soil Organic Carbon (SOC, g/kg), Total Nitrogen (TN, g/kg), Alkaline Hydrolysable Nitrogen (AN, mg/kg), Ammonium Nitrogen (NH_4_^+^-N, mg/kg), Nitrate Nitrogen (NO_3_^−^-N, mg/kg), Available Phosphorus (AP, mg/kg), Available Potassium (AK, mg/kg), Carbon to Nitrogen ratio (C/N ratio), Water Content (WC), and pH. To ensure the precision of the measurements, each sample underwent three replicates of parallel measurements.

The content of Soil Organic Carbon (SOC) was quantified using the potassium dichromate (K_2_Cr_2_O_7_) external heating method (Xue et al., 2014). Total Nitrogen (TN) levels were determined employing the Kjeldahl method (Saez-Plaza et al., 2013). The concentration of Alkaline Hydrolysable Nitrogen (AN) in the soil was ascertained through the alkaline diffusion method (Roberts et al., 2009). The amounts of Ammonium Nitrogen (NH_4_^+^-N) and Nitrate Nitrogen (NO_3_^−^-N) were measured after extraction with potassium chloride (KCl), followed by analysis using a flow injection analyzer (Li et al., 2012). The level of Available Phosphorus (AP) was determined post-digestion with sulfuric acid (H_2_SO_4_) and perchloric acid (HClO_4_) (Cade Menun and Lavkulich, 1997). The concentration of Available Potassium (AK) was measured using a flame photometer (Banerjee and Prasad, 2020). The Carbon to Nitrogen ratio (C/N ratio) was calculated based on the ratio of organic carbon content to total nitrogen content, and Water Content (WC) was determined via the direct drying method.

### Statistical analyses

The data analysis for this article was performed using the R programming language (version 4.3.3). For correlation analyses, we employed the corr.test function from the “psych” package (version 2.1.9 or similar), ensuring the assessment of relationships between soil properties and microbial community composition was statistically sound. Variance among groups was analyzed using the aov function, a core feature of base R for conducting Analysis of Variance (ANOVA), facilitating a comprehensive examination of differences across varied treatment levels.

In exploring the complex interactions within bacterial communities, Redundancy Analysis (RDA) and non-metric Multidimensional Scaling (NMDS) were conducted using functionalities from the “vegan” package (version 2.6–4). Visualization of our findings was enhanced by the “ggplot2” package (version 3.5.0). To further refine our figures, we incorporated aesthetic enhancements and custom visual elements using additional packages: “ggalt” (version 0.4.0) for alternative coordinate systems and statistical transformations, “ggpmisc” (version 0.5.5) for incorporating textual annotations and regression equation displays, and “ggsci” (version 3.0.3) for applying scientifically-themed color palettes. The “ggpubr” package (version 0.6.0) was also utilized for adding trend regression lines and creating publication-ready plots. Additionally, we used the “ggcor” package (version 0.9.4.3) to create and visualize correlation matrices, further enhancing our data presentation and interpretation.

## Results

### Two-way ANOVA of soil environmental nutrition factors

A two-way analysis of variance (ANOVA) was conducted to examine the effects of fire severity and soil layer on soil environmental nutrition factors, with the results presented in Table 2.

**Table 2:** Impact of severity and layer on soil environmental nutrition factors.

	SOC	TN	NH_4_^+^-N	NO_3_^−^-N	AN	AP	AK	C/N ratio	WC	pH
Severity	0.096	0.202	0.070	0.052	0.066	0.431	0.047^*^	0.533	0.470	0.001^**^
Layer	0.004^**^	0.013^*^	0.252	0.284	0.009^**^	0.573	0.030^*^	0.073	0.016^*^	0.882
Severity*Layer	0.957	0.980	0.870	0.573	0.934	0.655	0.938	0.938	0.646	0.994

Significance levels are denoted as follows: *Indicates *p* < 0.05, suggesting statistical significance; **indicates *p* < 0.01, denoting high statistical significance. These markers highlight the impact of severity and layer on various parameters within the analyzed ecosystem, based on the outcomes of a two-way ANOVA.

The summarized results from the two-way ANOVA illuminate the significant impacts of fire severity and soil layer on key soil and ecological parameters, emphasizing *p*-values to determine statistical significance. The analysis reveals that the layer has a statistically significant effect on SOC, TN, and AN with *p*-values of 0.004, 0.013, and 0.009, respectively, suggesting a pronounced influence of stratification on these properties. Similarly, severity significantly alters soil pH, as indicated by a *p*-value of 0.001, pointing to noteworthy changes in acidity linked to varying severity levels.

Both severity and layer significantly influence AK, as reflected by p-values of 0.047 and 0.030, highlighting their roles in the dynamics of potassium availability. Additionally, the layer also significantly affects the C/N ratio and water content (WC), with *p*-values of 0.073 and 0.016, respectively, indicating the importance of soil stratification in these parameters. However, the interaction between severity and layer does not show statistically significant effects on the evaluated parameters, suggesting that the impacts of these factors on the studied variables are independent rather than combined.

### Relationship between bacterial α-diversity and soil environmental factors

**Figure 2:**
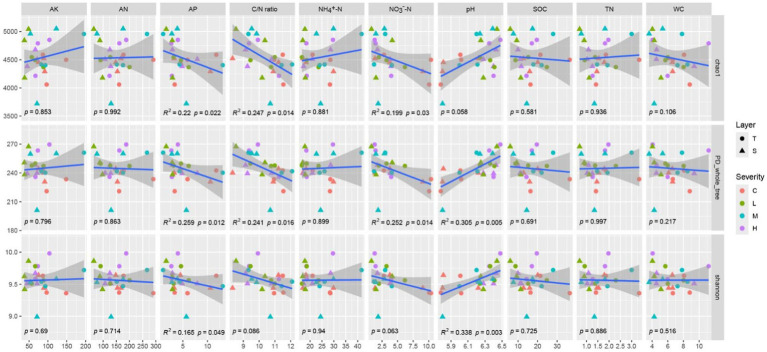
Correlation between α-diversity and soil environmental factors the gray shading represents the 95% confident interval for regressions. α-Diversity includes chao1, PD_whole_tree, Shannon index. Soil environmental factors includes Soil Organic Carbon (SOC), Total Nitrogen (TN), Ammonium Nitrogen (NH_4_^+^-N), Nitrate Nitrogen (NO_3_^−^-N), Alkaline Hydrolysable Nitrogen (AN), Available Phosphorus (AP), Available Potassium (AK), Carbon/Nitrogen (C/N) ratio, Water Content (WC), and pH. “C” stands for the control test, “L” represents light severity, “M” for moderate severity, and “H” for high severity of fire impact.

The Chao1 index demonstrates significant negative correlations with AP, C/N ratio, and NO_3_^−^-N (*p* < 0.05). Similarly, the PD_whole_tree index is significantly negatively correlated with AP, C/N ratio, and NO_3_^−^-N (*p* < 0.05), and shows a highly significant positive correlation with pH (*p* < 0.01). The Shannon index exhibits a significant negative correlation with AP and a highly significant positive correlation with pH (*p* < 0.01). The variations in other indicators do not significantly impact α-diversity (Figure 2).

### Relative abundances of dominant phyla under different fire severity levels

**Figure 3:**
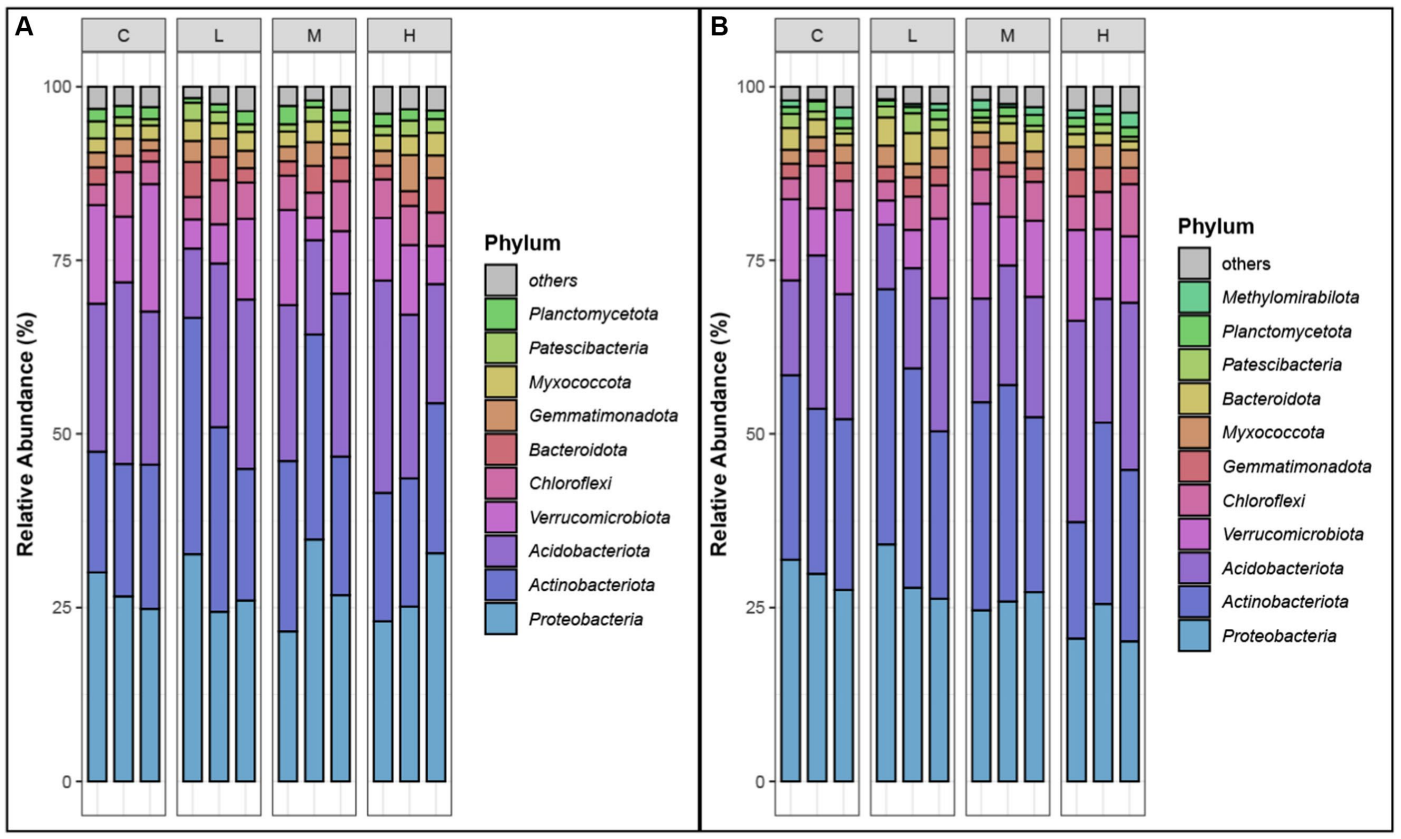
Relative abundances of dominant phyla. **(A)** top-layer soil. **(B)** Sub-layer soil. Phyla are selected as dominant phyla if their relative abundance is >1% in at least one sample type at each site. “C” stands for the control test, “L” represents light severity, “M” for moderate severity, and “H” for high severity of fire impact.

The analysis of the relative abundances of bacterial phyla in both the top soil layer (0–10 cm) and the sublayer (10–20 cm) across different fire severity levels (Control, Light, Moderate, High) revealed significant shifts in microbial communities (Figure 3). Dominant phyla, identified by having relative abundances greater than 1% in at least one sample type at each site (Zhang et al., 2021).

*Proteobacteria* consistently dominated in all conditions, with relative abundances ranging from 20.14 to 34.82%, indicating strong resilience. *Acidobacteriota* showed increased abundances in high severity areas, particularly in the sublayer, while *Actinobacteriota* thrived in light and moderate severity areas, reaching up to 36.73%. *Verrucomicrobiota* were more prevalent in control samples and decreased significantly in fire-impacted soils. *Chloroflexi* and *Bacteroidota* displayed increased abundance in moderate and high severity areas, respectively. Other phyla such as *Patescibacteria*, *Gemmatimonadota*, *Myxococcota*, and *Planctomycetota* also demonstrated notable changes in response to fire severity. These shifts highlight the impact of fire on soil microbial composition, with different phyla showing varying levels of adaptability to fire-induced changes.

### Correlation analysis of soil environmental factors and dominant bacterial phyla

**Figure 4:**
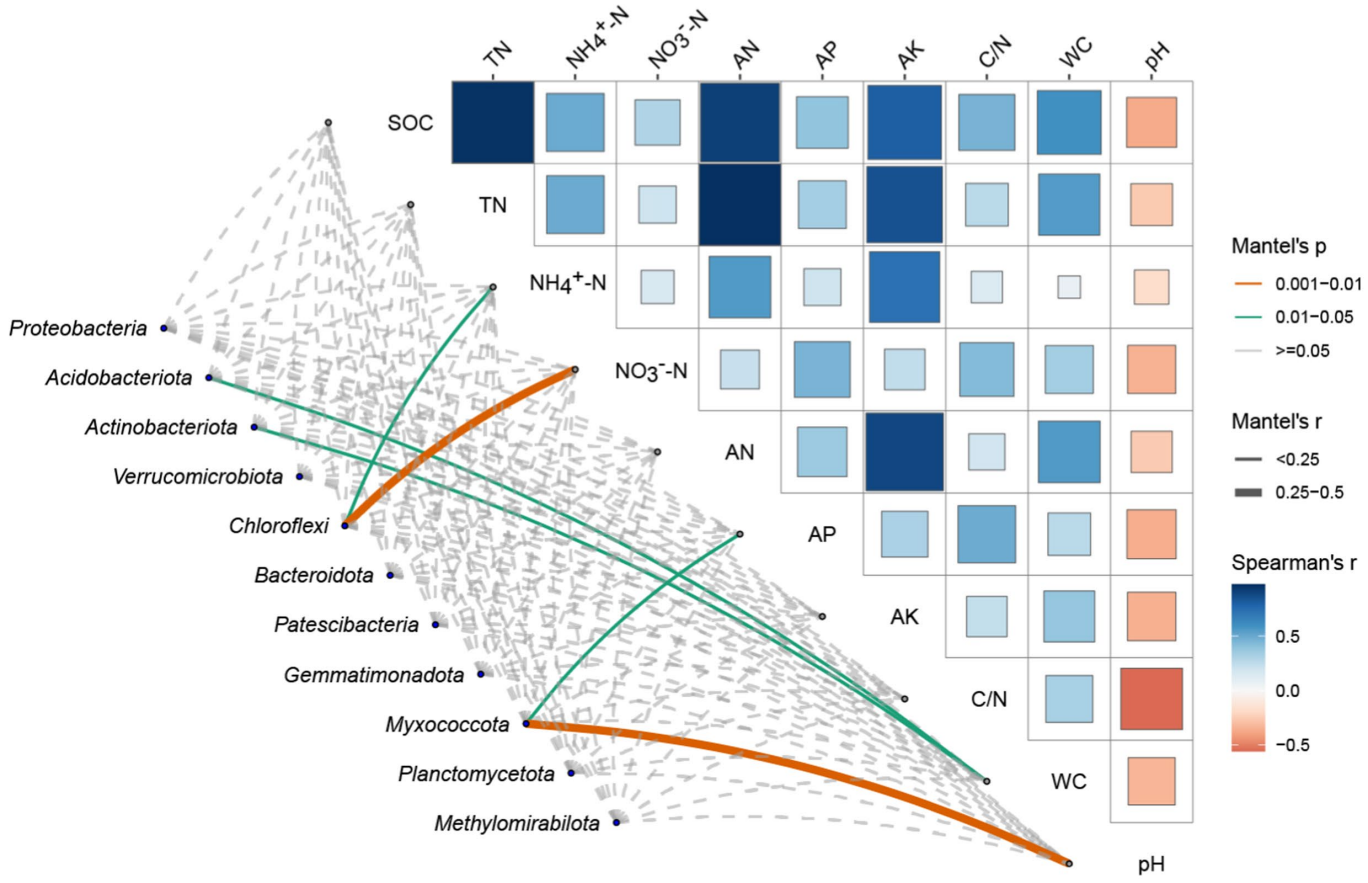
Spearman correlation and mantel test analysis between soil environmental factors and dominant bacterial phyla soil environmental factors includes Soil Organic Carbon (SOC), Total Nitrogen (TN), Ammonium Nitrogen (NH_4_^+^-N), Nitrate Nitrogen (NO_3_^−^-N), Alkaline Hydrolysable Nitrogen (AN), Available Phosphorus (AP), Available Potassium (AK), Carbon/Nitrogen (C/N) ratio, Water Content (WC), and pH. The upper triangular matrix in Figure 4 displays the Spearman correlation coefficients between the environmental factors, with colors representing the strength and direction of the correlations (blue for negative, red for positive). The size of the squares is proportional to the magnitude of the correlation coefficients. Overlaying the correlation matrix, lines represent significant relationships identified by the Mantel test, where line color indicates the *p*-value category (<0.001, 0.001–0.01, 0.01–0.05, ≥0.05), line thickness represents the Mantel’s *r* value (<0.25, 0.25–0.5, ≥0.5), and line type (solid or dashed) corresponds to the *p*-value significance levels.

We illustrates the Spearman correlation matrix and Mantel test results between selected soil environmental factors and dominant bacterial phyla in *P. tabulaeformis* forest soils (Figure 4).

By analyzing the correlation matrix of soil environmental factors in the top right corner, it is evident that soil organic carbon content is positively correlated with various soil environmental factors, especially with total nitrogen content and alkaline hydrolysable nitrogen (AN). Furthermore, soil nitrogen content is positively correlated with available potassium (AK). Notably, soil pH exhibits a negative correlation with multiple soil environmental factors. From the association between the two matrices, it is clear that soil pH and available phosphorus (AP) significantly influence the abundance of the phylum Myxococcota. Soil water content (WC) significantly affects the abundances of *Acidobacteriota* and *Actinobacteriota*. Additionally, ammonium nitrogen (NH_4_^+^-N) and nitrate nitrogen (NO_3_^−^-N) jointly and significantly impact the abundance of the phylum Chloroflexi.

### Effects of different fire severities and soil environmental nutrition factors on microbial taxa

The adoption of Redundancy Analysis (RDA) over Canonical Correspondence Analysis (CCA) in this investigation was based on preliminary findings from a detrended correspondence analysis (DCA) performed using the decorana function in the vegan package. The DCA results, revealing that all axes’ lengths were under 3.0, suggested a linear relationship between the environmental variables and species data, thus indicating the appropriateness of RDA for our dataset comprising soil nutrient data and microbial community compositions in the topsoil (0-10 cm) and subsoil layers (10–20 cm).

**Figure 5:**
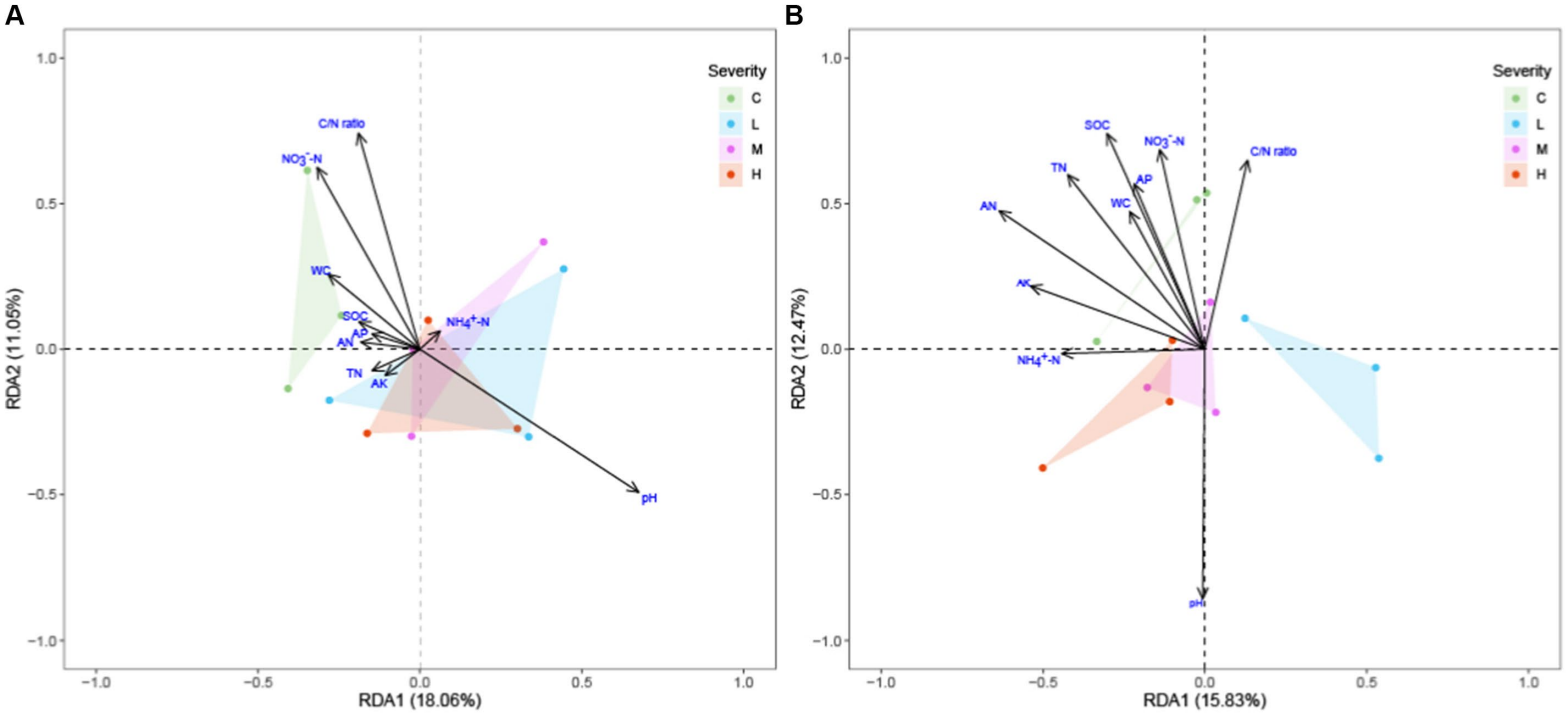
RDA of soil microbial community structure to soil environmental factors across different fire severities and soil depths soil environmental factors includes: Soil Organic Carbon (SOC), Total Nitrogen (TN), Ammonium Nitrogen (NH_4_^+^-N), Nitrate Nitrogen (NO_3_^−^-N), Alkaline Hydrolysable Nitrogen (AN), Available Phosphorus (AP), Available Potassium (AK), Carbon/Nitrogen (C/N) ratio, Water Content (WC), and pH. “C” stands for the control test, “L” represents light severity, “M” for moderate severity, and “H” for high severity of fire impact. **(A)** top-layer soil **(B)** sub-layer soil.

Our RDA exploration (Figure 5) into the effects of fire severity on ecosystem composition across the topsoil and subsoil layers revealed significant differential impacts. In the topsoil, pH and C/N ratio emerged as the dominant environmental gradients influencing species distribution, with the vector of pH delineating its pivotal role in modulating species compositional variability. Remarkably, in the sublayer soil, alongside SOC, TN, and NO_3_^−^-N, the vector for pH was notably extended, underscoring its critical importance in influencing ecosystem variability—a finding that mirrors its significant role in the topsoil layer. The distribution of sample points highlighted the nuanced effects of fire severity on ecosystems, with distinct clustering patterns for different fire severity groups evident in both soil layers. This pattern underscores significant ecosystem distinctions under varying fire regimes, with the subsoil layer’s analysis further illuminating the profound influence of pH in modulating these effects.

### Differences in microbial communities under the influence of different fire intensities

**Figure 6:**
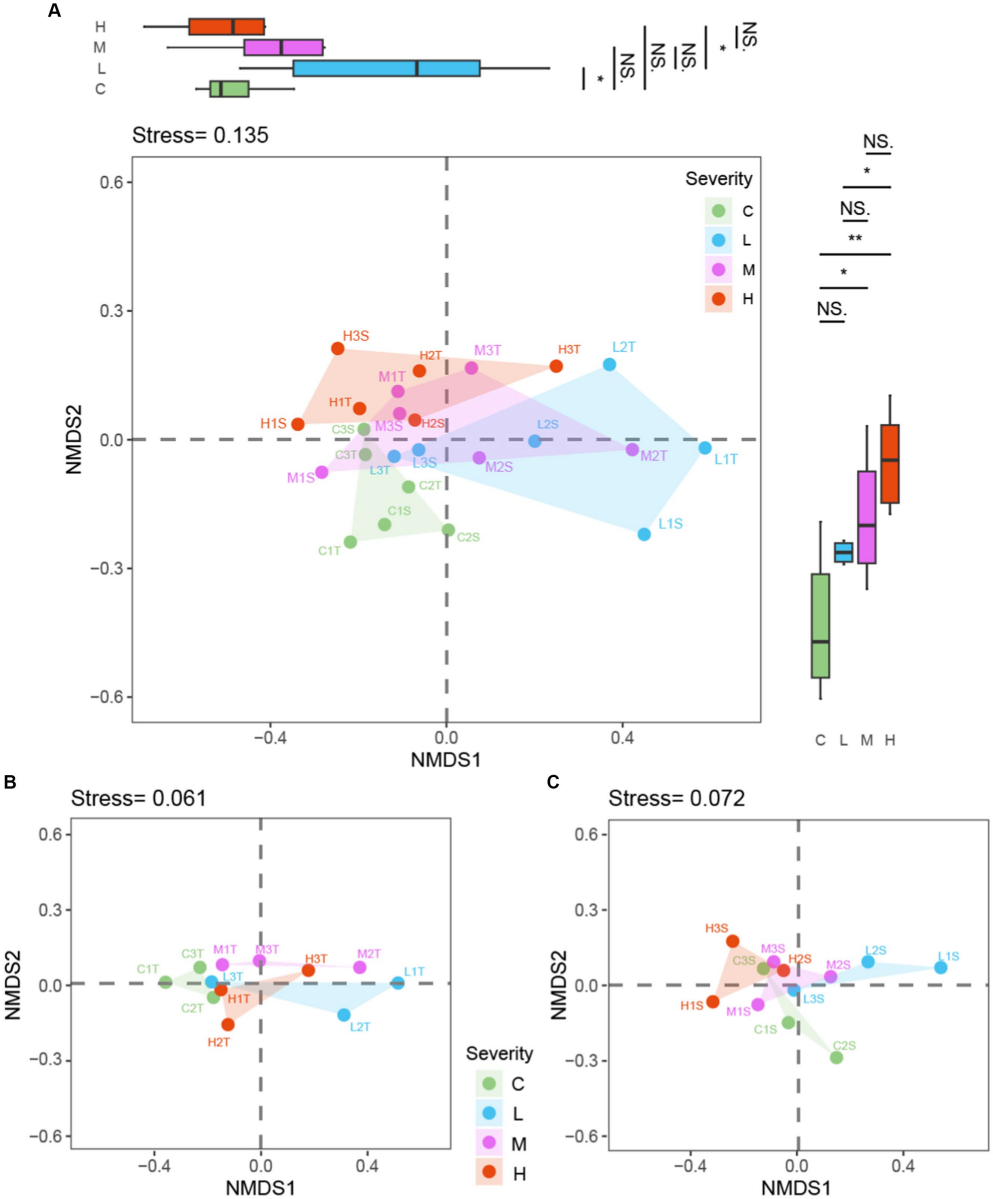
NMDS analysis by fire severity: combined dataset and Soil Layer Comparison “C” stands for the control test, “L” represents light severity, “M” for moderate severity, and “H” for high severity of fire impact. “T” stands for top-layer and “S” stands for sub-layer. **(A)** both layers soil, **(B)** top-layer soil, **(C)** sub-layer soil.

The Non-metric Multidimensional Scaling (NMDS) analysis, employing Bray–Curtis dissimilarity, was applied to assess how fire severity influences microbial community composition across various soil layers. This analysis encompassed a collective dataset along with individual analyses for topsoil (0–10 cm) and subsoil (10–20 cm) layers. Principal Coordinates (PC) analysis was conducted on each NMDS axis to further elucidate the distinctions among fire severity groups (Figure 6).

Within the NMDS analysis presented in Figure 6, the notations used denote specific conditions and layers: “C” stands for the control test, “L” represents light severity, “M” for moderate severity, and “H” for high severity of fire impact. Additionally, “T” signifies the top layer of soil (0–10 cm), and “S” indicates the sub-layer (10–20 cm). For instance, “C1T” refer to the top-layer sample from the first control test plot, with similar logic applied to other labels, facilitating a detailed interpretation of the microbial community composition in response to varying degrees of fire severity across different soil depths.

The combined dataset NMDS analysis, yielding a stress value of 0.135, demonstrated a significant separation among microbial communities corresponding to different fire severities. PC analysis on the NMDS1 axis identified significant differences between Control (C) and Light (L) burn conditions (*p* < 0.05), as well as between Light (L) and High (H) burn conditions (*p* < 0.05). The NMDS2 axis revealed notable disparities, with significant differences between Control (C) and Moderate (M) (*p* < 0.05), Control (C) and High (H) (*p* < 0.01), and Light (L) and High (H) (*p* < 0.05).

Focusing exclusively on the topsoil layer, the NMDS and subsequent PC analyses uncovered patterns similar to those observed in the combined dataset. This layer-specific analysis provides insight into the distinct ways in which the surface microbial communities respond to fire severity, suggesting that topsoil microbes might be particularly sensitive to the variations in fire severity. The subsoil layer NMDS and PC analyses offer a complementary perspective, indicating that subsoil microbial communities also exhibit distinct responses to fire severity, albeit with some nuanced differences from the topsoil responses. These findings highlight the importance of considering soil depth in ecological studies of fire effects, as the subsoil microbial communities demonstrate resilience and distinct patterns of change in response to fire.

The comprehensive approach, integrating NMDS analyses with PC findings across combined, topsoil, and subsoil datasets, elucidates the multifaceted impacts of fire severity on soil microbial ecosystems. Each layer’s analysis, reinforced by PC analysis findings, underscores the significant, yet complex, influence of fire severity on microbial community dynamics. Specifically, the PC analyses bring statistical rigor to the NMDS-derived observations, confirming the ecological relevance of the detected patterns among different fire severity groups and soil depths.

## Discussion

### Interactions of fire severity and soil depth on soil properties

The significant differences in soil organic carbon (SOC) underscore the role of soil depth in regulating SOC dynamics. Our research found that higher fire severity and deeper soil layers lead to variations in SOC levels. As a key indicator of soil quality and ecosystem function, changes in SOC are closely linked to the capacity for microbial decomposition of organic matter, the input of plant residues, and alterations in the soil environment post-fire, such as changes in soil temperature and moisture (Wiesmeier et al., 2019). Additionally, variations in SOC may also relate to changes in soil particle structure and the soil’s capacity to retain moisture, factors that collectively influence the rate of accumulation and decomposition of organic carbon. Yang et al. (2024) observed similar patterns in SOC dynamics following forest fires, supporting our findings and highlighting the complex interplay between fire-induced environmental changes and soil carbon storage mechanisms.

The significant differences in alkaline hydrolyzable nitrogen (AN), an important indicator of soil fertility, highlight the distribution of plant-available nitrogen sources across different soil layers. Our research found that soil layer significantly influences AN level, with a *p*-value of 0.009, suggesting that soil depth is a crucial factor in determining nitrogen availability. This is in contrast to the impact of fire severity on AN, which was not significant (*p* = 0.066) according to the two-way ANOVA results. Ahmad et al. (2021) indicated that variability in AN can be influenced by plant root systems’ uptake of nitrogen, microbial conversion of organic nitrogen, and the impact of fire on soil pH (Ahmad et al., 2021). While our findings align with the importance of these factors, they also suggest that soil depth plays a more significant role than fire severity in influencing AN dynamic. Eckdahl et al. (2023) noted similar effects of fire on microbial community structure, which could indirectly affect AN level (Eckdahl et al., 2023), but our results highlight that depth-related microenvironmental conditions are more pivotal. Moreover, changes in AN in our study are related to microenvironmental conditions associated with soil depth, such as moisture and oxygen availability. This is consistent with the findings of Martin et al. (2022) and Zhao et al. (2022), who reported that soil depth-related microenvironmental conditions significantly impact nitrogen dynamics (Martin et al., 2022; Zhao et al., 2022). Ling et al. (2021) emphasized that post-fire shifts in soil nitrogen availability are closely tied to changes in soil microbial processes and environmental conditions (Ling et al., 2021). Our research supports this by demonstrating the critical role of soil depth in shaping soil nitrogen dynamics, emphasizing the need for considering soil stratification in managing soil fertility post-fire.

The marked impact of fire severity on soil pH, which is statistically significant (*p* = 0.001), highlights the role of fire-induced changes in soil chemistry. Our research found that fire severity significantly altered soil pH, likely due to the deposition of ash that results in a liming effect (Sun et al., 2020). Some researchers suggested that ash deposition raises soil pH by adding basic cations (Marcotte et al., 2022). However, our study shows that this increase in pH also significantly influences nutrient solubility and microbial community composition, indicating a broader impact of fire severity on soil health. Additionally, both fire severity (*p* = 0.047) and soil layer (*p* = 0.030) significantly affect available potassium (AK) levels. Pesini et al. (2024) indicated that potassium mobility and plant absorption vary with soil depth (Pesini et al., 2024), our findings further demonstrate that fire severity alters AK availability through changes in soil pH and the mineralization of potassium-rich organic matter. This suggests that the interaction between fire severity and soil stratification plays a crucial role in post-fire nutrient dynamics.

The lack of significant differences in Nitrate Nitrogen (NO_3_^−^-N) and Ammonium Nitrogen (NH_4_^+^-N) across soil layers in our study suggests that the biogeochemical cycling characteristics of these nitrogen forms lead to uniform distribution. NO_3_^−^-N, being more soluble and mobile, likely distributes more uniformly across the soil profile, while NH_4_^+^-N, which tends to be more readily adsorbed by soil particles, shares this uniform distribution trait. Almaz et al. noted that the physicochemical properties of these nitrogen forms contribute to their uniform distribution, which aligns with our findings (Almaz et al., 2023). Additionally, the equilibrium between nitrification and denitrification processes likely maintains relative stability in nitrogen distribution across layers. Xue et al. highlighted the role of these processes in stabilizing nitrogen forms in the soil, which our results support (Xue et al., 2022). Choromanska et al. found that soils previously exposed to fire had low NH_4_^+^-N concentrations and high NO_3_^−^-N concentrations, indicating that fire can significantly alter nitrogen forms (Choromanska and DeLuca, 2002). In contrast, DeBano et al. reported little change in NO_3_^−^-N and NH_4_^+^-N when litter and soil were moist during a burn (DeBano et al., 1979). Our research aligns with DeBano et al.’s findings, showing no significant impact of fire severity on NO_3_^−^-N and NH_4_^+^-N levels. Six years after the wildfire, the uniform distribution of NO_3_^−^-N and NH_4_^+^-N in our study suggests a natural stability in nitrogen cycling and the resilience of microbial communities within the ecosystem. Bouskill et al. emphasized the gradual restoration of soil chemistry and microbial communities to pre-fire conditions over the long term (Bouskill et al., 2022). Our findings reflect this gradual recovery, indicating that post-fire soil chemistry and microbial communities initially undergo rapid changes but eventually stabilize, including the distribution of nitrogen forms and the maintenance of biogeochemical cycling processes.

Our analysis reveals no significant interaction effects between fire severity and soil layer on other soil properties. This observation might reflect several underlying ecological dynamics and soil physicochemical processes. The mechanisms through which fire severity influences soil—primarily through the direct alteration of soil chemistry via combustion and ash deposition—operate distinctly from the gradual influences of soil depth, which include organic matter accumulation and microbial activity variations. This suggests that the specific effects of fire severity and soil depth may not synergistically interact to further modify soil properties beyond their independent impacts.

Furthermore, soils possess inherent buffering capacities that can mitigate the alterations induced by fire and variations in soil depth, potentially contributing to the observed stability across soil layers (Nazir et al., 2024). The spatial and temporal variability inherent in post-fire ecosystems could also mask potential interaction effects, as the influence of fire severity and soil depth may manifest differently across various ecological contexts and recovery timelines. Additionally, the resilience of ecosystems, especially those adapted to periodic fire disturbances, may play a role in quickly re-establishing equilibrium within soil properties, negating the expected interactive effects.

### Impact of soil properties on α-biodiversity

Our findings indicate a negative correlation between α-diversity indices and available phosphorus (AP), C/N ratio, and nitrate nitrogen (NO_3_^−^-N), alongside a positive correlation with soil pH. These correlations underscore the intricate balance between nutrient availability and microbial diversity within the soil ecosystem. The negative correlation with AP, C/N ratio, and NO_3_^−^-N suggests that high nutrient levels might lead to the dominance of specific microbial taxa that are more efficient at utilizing these resources, potentially outcompeting other species and thus reducing overall diversity (Huang et al., 2023). In Huang et al.’s (2023) study, various data analyses have demonstrated that an increase in nitrogen elements leads to a decrease in community biodiversity. Conversely, the positive correlation with soil pH points to the importance of maintaining a balanced pH level to support a diverse microbial community. The variation in microbial species’ tolerance and optimum pH ranges suggests that a neutral to slightly alkaline soil environment could foster a broader range of microbial life (Ng et al., 2023), contributing to ecosystem resilience and function. This finding emphasizes the potential benefits of pH adjustments in ecological restoration projects, aiming to enhance microbial diversity and thereby ecosystem services.

It is evident that fire severity has an extremely significant impact on soil pH, with a *p*-value of 0.001. Furthermore, Figure 4 shows that soil pH is negatively correlated with various soil properties, which is consistent with the findings of Qin and Liu (2021). Our exploration into the effects of wildfires on *P. tabulaeformis* forests reveals a critical link between soil pH, fire severity, and the subsequent microbial community composition and diversity. Wildfires fundamentally alter soil chemistry, primarily through the combustion of organic matter and the deposition of ash, which typically results in an increase in soil pH levels. This ash deposition, rich in basic cations, effectively acts as a liming agent, raising the pH of the soil surface and affecting deeper layers through nutrient leaching and percolation.

Our study revealed a highly significant negative correlation between soil pH and the carbon-to-nitrogen (C/N) ratio, particularly in the context of post-wildfire conditions. This relationship can be explained by the influence of slightly increased soil pH on organic matter decomposition and nutrient mineralization processes following a wildfire. Even modest increases in soil pH, which often result from the deposition of ash, can enhance microbial activity. This leads to increased decomposition rates of organic matter, reducing the C/N ratio by promoting the mineralization of organic carbon and nitrogen into more readily available inorganic forms (Sun et al., 2017). Other researchers observed similar patterns, noting that higher pH levels, even within a neutral to slightly acidic range, can lead to a decrease in the C/N ratio due to the enhanced breakdown of organic materials (Tahmasbian et al., 2019; Zema and Lucas-Borja, 2023). Our findings align with this, suggesting that the elevated pH following a wildfire accelerates the decomposition process, thereby reducing the C/N ratio. This reduction in the C/N ratio post-wildfire can have profound effects on nutrient cycling and availability, potentially influencing plant growth and microbial community structure by increasing the availability of nitrogen in more accessible forms for both plants and microbes.

The elevated soil pH post-wildfire creates a higher pH environment that significantly impacts the soil’s microbial landscape and can promote the growth of certain microbial taxa that are better adapted to higher pH levels (Marin et al., 2021). Moreover, the increase in soil pH can enhance the availability of nutrients that were previously bound in acidic conditions (Lu et al., 2022), further influencing microbial community dynamics. This suggests that post-fire soil conditions, mediated by increased pH, provide a conducive environment for a wider range of microbial taxa, enhancing biodiversity. Adjusting soil pH to optimal levels for microbial diversity and ecosystem function could be key to promoting post-fire recovery and resilience (Perez-Valera et al., 2020). These interventions, tailored to the specific conditions and needs of affected forest soils, could markedly enhance the resilience of forest ecosystems to future disturbances and aid in their long-term recovery.

The highly significant correlation between pH and α-diversity, can be attributed to the adaptive capabilities of microbial communities to fluctuating moisture levels and the overriding influence of pH on microbial metabolism. Unlike pH, which directly impacts the metabolic activities and growth conditions of microbes, variations in water content may not significantly stress microbial communities in environments where moisture rarely limits microbial activity (Wang et al., 2022). This suggests that in the context of our study area, pH plays a more critical role than water content in determining microbial community diversity. The specific impacts of soil physical properties like water content on microbial diversity may be nuanced and influenced by regional climate conditions, soil texture, and the temporal stability of moisture levels (Rocabruna et al., 2024).

Our results show that soil pH and available phosphorus (AP) significantly influence the abundance of the phylum *Myxococcota*. These findings are consistent with the conclusions of Tian et al. (2023). This may be attributed to the role of pH in regulating microbial activity and nutrient availability. Higher pH levels can enhance the solubility of certain nutrients, making them more accessible to microbes that thrive in alkaline conditions. Similarly, available phosphorus is a crucial nutrient that can limit or promote microbial growth depending on its concentration in the soil. Soil water content (WC) significantly affects the abundances of *Acidobacteriota* and *Actinobacteriota*. It can be explained by the dependency of these microbial groups on moisture levels for their metabolic processes. Adequate water content in the soil creates a favorable environment for these bacteria, supporting their growth and activity (Tian et al., 2023). Additionally, ammonium nitrogen (NH_4_^+^-N) and nitrate nitrogen (NO_3_^−^-N) jointly and significantly impact the abundance of the phylum *Chloroflexi*. This is maybe because both ammonium and nitrate are essential nitrogen sources for microbial metabolism. Their availability can significantly affect the growth and distribution of nitrogen-utilizing microbial taxa. Ammonium nitrogen (NH_4_^+^-N) and nitrate nitrogen (NO_3_^−^-N) jointly and significantly impact the abundance of the phylum *Chloroflexi*, which is consistent with the findings of Nie et al., the correlation likely arises because both ammonium and nitrate serve as critical nitrogen sources for microbial metabolism (Nie et al., 2018). The availability of these nitrogen forms can substantially influence the growth and distribution of nitrogen-utilizing microbial taxa, thereby affecting the overall microbial community structure. The presence of NH_4_^+^-N and NO_3_^−^-N provides essential nutrients that facilitate the metabolic processes of *Chloroflexi*, highlighting the importance of nitrogen availability in regulating microbial abundance and diversity within soil ecosystems.

### The moderating role of soil depth in the effects of fire

Six years following the wildfire in *P. tabulaeformis* forests, our integrated analyses using Redundancy Analysis (RDA), Non-metric Multidimensional Scaling (NMDS), and Principal Coordinates (PC) analysis have illuminated the complex interplay between soil depth, fire severity, and their combined effects on microbial community structures and soil physicochemical properties. RDA highlighted how environmental factors such as pH, SOC, and TN influence microbial distribution across soil depths and fire severities. NMDS visualized the distinct separation of microbial communities based on fire severity and soil depth, showing clear clustering patterns. PC analysis further confirmed these distinctions, emphasizing the significant differences in microbial community compositions across varying fire severities. Together, these analyses underscore the nuanced role of soil depth in shaping post-fire microbial recovery, providing a comprehensive view of forest ecosystem resilience in response to fire disturbances.

The topsoil layer, being the primary interface with environmental conditions, undergoes a rapid microbial adjustment towards a new equilibrium post-fire. This swift recovery is attributed to its direct exposure to environmental changes, including the influx of nutrients and organic matter (Song X et al., 2021). Such exposure not only facilitates rapid recolonization and microbial succession but also subjects the topsoil to more pronounced natural weathering and external disturbances. Despite clear distinctions in microbial community composition between burned and unburned plots, variations among fire severities within the topsoil layer are less pronounced, suggesting a threshold effect where initial fire disturbances set the stage for microbial community adjustment (Ling et al., 2021), with minimal additional impact from further variations in fire severity. This pattern is refined by NMDS and PC analyses, which highlight significant separations in microbial community structures across different fire severities, indicating that even minimal burn conditions deviate markedly from unburned controls. In contrast, the subsoil layer follows a divergent path of microbial succession, characterized by slower recovery and deeper ecological shifts (Li et al., 2023). The reduced direct impact of fire and subsequent environmental changes on this layer, coupled with the nutrient transfer dynamics from the topsoil, points to a prolonged period of ecological adjustment and microbial community reshaping (Ling et al., 2021). The nutrient seepage from the topsoil acts as a buffering mechanism, mitigating the reduced exposure of the subsoil to post-fire changes and supporting a gradual and sustained microbial recovery (Verrone et al., 2024). This process emphasizes the importance of considering vertical nutrient dynamics and the layered impact of wildfire on forest ecosystems.

Furthermore, the comparative analysis of topsoil and subsoil layers reveals the moderating role of soil depth on the ecological impacts of fire (Xu S et al., 2022). Soil depth not only influences the immediate post-fire microbial community composition but also plays a crucial role in the long-term ecological recovery and stabilization after a wildfire (Qin and Liu, 2021; Yang et al., 2024). The observed microbial community structures across soil depths suggest that post-fire restoration and management strategies should be specifically tailored to address the distinct needs and dynamics of both topsoil and subsoil layers, thereby enhancing the overall recovery and resilience of the ecosystem.

## Conclusion

This study investigated the impact of anthropogenic wildfires on soil microenvironment heterogeneity and bacterial community structure in *P. tabulaeformis* forests in North China, focusing on varying fire intensities and soil depths 6 years post-fire. Our findings demonstrate the long-term effects of wildfires on soil physicochemical properties and microbial diversity, with soil pH playing a pivotal role in shaping post-fire microbial community dynamics. Significant differences in soil bacterial communities were observed between burned and unburned areas, indicating lasting impacts of wildfires. While fire intensity showed minimal impact on topsoil bacterial communities, significant changes were evident in subsoil bacterial communities, suggesting a differential ecological recovery process across soil layers. Moreover, as soil pH increased, bacterial community diversity also increased, indicating that wildfires indirectly influence bacterial communities by increasing soil pH, thereby enhancing species diversity.

Future research should focus on long-term monitoring to further elucidate the temporal dynamics of soil microbial recovery and nutrient cycling following wildfires. Investigating the role of specific microbial taxa in ecosystem resilience and their functional contributions under varying fire regimes could provide deeper insights. Additionally, exploring the potential of soil amendments and management practices aimed at optimizing soil pH and nutrient availability may enhance post-fire recovery processes. Such studies will be crucial for developing targeted forest management strategies that mitigate the adverse effects of wildfires and promote sustainable ecosystem recovery.

## Data availability statement

The datasets presented in this study can be found in online repositories. The names of the repository/repositories and accession number(s) can be found at: https://www.ncbi.nlm.nih.gov/, PRJNA1097343.

## Author contributions

GL: Data curation, Formal analysis, Investigation, Methodology, Software, Visualization, Writing – original draft, Conceptualization. ZG: Writing – review & editing, Investigation. XL: Writing – review & editing, Investigation, Resources, Validation. BL: Data curation, Writing – review & editing, Funding acquisition, Investigation, Project administration.

## Footnotes

^1^ http://qiime.org/
